# Efficiency of *Bacillus thuringiensis* and *Bacillus cereus* against *Rhynchophorus ferrugineus*

**DOI:** 10.3390/insects13100905

**Published:** 2022-10-05

**Authors:** Mohsen Mohamed Elsharkawy, Mustafa Almasoud, Yasser Mohamed Alsulaiman, Rowida S. Baeshen, Hayam Elshazly, Roqayah H. Kadi, Mohamed M. Hassan, Rady Shawer

**Affiliations:** 1 Department of Agricultural Botany, Faculty of Agriculture, Kafrelsheikh University, Kafr El-Sheikh 33516, Egypt; 2 Palms and Dates Center, Ministry of Environment, Water and Agriculture, Al Ahsa 31982, Saudi Arabia; 3 Department of Biology, Faculty of Science, University of Tabuk, Tabuk 71421, Saudi Arabia; 4 Department of Biology, Faculty of Science & Arts-Scientific Departments, Qassim University, Buraidah 52571, Saudi Arabia; 5 Department of Zoology, Faculty of Science, Beni-Suef University, Beni Suef 62521, Egypt; 6 Department of Biology, Faculty of Science, University of Jeddah, Jeddah 21959, Saudi Arabia; 7 Department of Biology, College of Science, Taif University, Taif 21944, Saudi Arabia; 8 Department of Plant Protection, Faculty of Agriculture (Saba Basha), University of Alexandria, Alexandria 21531, Egypt

**Keywords:** *Bacillus thuringiensis*, *Bacillus cereus*, Red Palm Weevil, entomopathogenic bacteria, rhizosphere bacteria, date palm

## Abstract

**Simple Summary:**

*Rynchophorus ferrugineus* Olivier, the red palm weevil (RPW), is a damaging insect that often severely infests palm trees. Since insecticides have a detrimental environmental impact and may lead to pesticide resistance, new biological control methods are needed. Bacteria from many different species affect the RPW larval growth, health, and immunity. The work evaluates the crosstalk between the RPW and rhizosphere bacteria. Four bacterial isolates, three belonging to *Bacillus cereus* and one belonging to *B. thuringiensis,* had a significantly higher impact on the mortality of the larvae and adults of the RPW than other bacteria that were tested did. The results emphasize the significant potential of developing new microbial resource-based management techniques for this pest.

**Abstract:**

The Red Palm Weevil (*Rhynchophorus ferrugineus* (Oliv.) (Coleoptera, Dryophthoridae) is a well-known palm tree pest that has caused enormous economic damage all over the globe. Insecticides are still the primary method of controlling this pest at this period. However, field populations of RPW have been shown to be resistant to pesticides. Using *Bacillus* spp. might be one of the options for controlling *R. ferruginous*. In this study, 23 species of *Bacillus* spp. were isolated from the rhizosphere of date palm trees in Al Ahsa Oasis, Saudi Arabia. The isolates were identified using 16S rRNA gene sequencing. *R. ferrugineus* larvae and adults were tested on sugarcane pieces that were treated with the *B. thuringiensis* strain PDC-AHSAA1 and *B. cereus* strains (PDC-AHSAA2, PDC-AHSA3 and PDC-AHSA4). The LC_50_ values for larvae and adults were quite low when they were compared with those of the other isolated strains. The *B. thuringiensis* strain PDC-AHSAA1 was more effective against both the larvae and adults. The determined LC_50_ values for *B. thuringiensis* ranged from 4.19 × 10^7^–3.78 × 10^9^. After 21 days, the data on larval mortality and body weight were evaluated. The surviving larvae that were treated with the bacterial isolates did not acquire a substantial weight. For the RPW larvae and adults, the mortality and corrected mortality death rates were increased by increasing the concentration of *B. thuringiensis*. In conclusion, *Bacillus*-treated diets negatively influenced the growth and development of the RPW. This research reported on the interaction between the RPW and the rhizosphere *Bacillus* spp. and highlighted the tremendous potential for the development of microbial resource-based control strategies for this pest.

## 1. Introduction

The *Rhynchophorus ferrugineus* (Olivier) is the most destructive and devastating pest of many palm plants and it originates from tropical Asia [1]. *R. ferrugineus*, which is known as the Red Palm Weevil (RPW), affects all palm trees including the date palm (*Phoenix dactylifera* L.) and the coconut (*Cocos nucifera* L.) as well as urban palmscapes (e.g., those that are dominated by the Canary Islands date palms) (*Phoenix canariensis* Chabaud) [2]. The direct losses in date palm production due to their infestation with RPWs reached $5.18 to $25.92 million [3]. It is common for female adults to deposit their eggs in tree wounds, fissures, and cracks. The larvae, which are the most destructive, travel into the palm trunk to create tunnels and enormous holes with damp-fermenting debris, therefore leading to frond deformity and palm death [4,5,6]. Adult RPWs can fly a very long distance, which is a distinct advantage that they have [7,8]. So far, RPWs have been reported in the Middle East, South Asia, and in the Mediterranean area [4,9]. There are costs that are associated with the removal and disposal of dead palms which have lowered property values and caused the degradation of recreational areas and urban wildlife habitats, which have all been results of RPW infestation [10]. None of the natural enemies to control the invasive species that have been transported from other locations were found to reach their full biological potential. Few natural pathogens are known for *R. ferrugineus*, and its chemical management, even in urban areas, is far from acceptable in the case of the RPW outbreak on *Phoenix dactylifera*. Prophylactic pesticide applications to protect palms from weevil infestation have resulted in harm and expenses. As a result, controlling the RPW infestation remains a big problem for researchers across the globe. 

The RPW is now being controlled by the use of exclusionary quarantines and pheromone traps. On the other hand, insecticide applications remain the most effective method of preventing palm weevil attacks. Insecticides, including carbamates, organophosphates, neonicotinoids, and phenylpyrazoles, for example, have been sprayed on foliage, applied to wounds, used as crown or soil drenches or injected into tree trunks or soil around the base of a tree trunk [10,11,12]. Resistance to these synthetic pesticides such as imidacloprid, chlorpyrifos, and lambda-cyhalothrin has been observed in the field populations of the RPW [13,14,15]. Biocontrol agents offer different benefits over chemical pesticides, such as environmental sustainability and minimal toxicity. Although researchers have utilized *Bacillus* species (*Brevibacillus laterosporus*, *B. aeruginosa, B. sphaericus*, and *B. megaterium*) against the RPW, none of these pathogens could be considered harmful due to their low pathogenicity [16]. The utilization of natural enemies as means of controlling these pests is an important option. *Metarhizium anisopliae*, *Beauveria bassiana*, *Steinernema carpocapsae*, *Serratia marcescens*, and *Bacillus thuringiensis* (Bt) have been demonstrated to be effective against the RPW [17,18,19,20,21,22,23]. It was interesting to explore the potential of date palm rhizosphere bacteria against the RPW.

Several different types of higher organisms inhabit the same ecosystem and may interact with each other, thereby giving the chance for the exchange between microorganisms and such animals and plants. As a result of their close interaction with plants and a wide range of plant-beneficial capabilities, rhizosphere bacteria have been the subject of extensive research. Bacterial pathogens are currently being studied for their possible utility in controlling pests. As an alternative to artificial chemicals, entomopathogenic bacteria exhibit considerable action selectivity and may be a viable option. Different pests may be controlled biologically using products that are based on *B. thuringiensis* [24]. Coleoptera is often targeted by Bt strains that have been isolated and characterized [1,25]. Evidence has emerged with reference to the possible pathogenicity against pests of various strains of *B. thuringiensis* [26,27]. After being exposed to *B. thuringiensis* in the lab, the RPW larvae showed decreased levels of boring activity and eating behavior [10]. The mortality of the RPW larvae that were exposed to *B. thuringiensis* subsp. *kurstaki* (Btk) and a polyhedrosis virus was 70% and 61%, respectively [28]. The field tests that have been conducted on these biological agents have so far shown modest success [29,30]. As a result of these findings, new microbes-based pest control techniques should be developed that are based on insect-associated microorganisms.

Currently, the only biological control agents that are available for the suppression of *R. ferrugineus* are entomopathogenic nematodes and *Beauveria bassiana* [19], and even with these, an adequate level of control is not always achieved, so the addition of entomopathogenic *Bacillus* to the RPW management strategies in urban countries would be most encouraged. The potential of Bt and *B. cereus* as biological control agents against the RPW larvae and adults was investigated which could be extended in the future to utilize these pathogens in management strategies.

## 2. Materials and Methods

### 2.1. Collection and Rearing of Rhyncophorus ferrugineus

Larvae and adults were gathered from infested palms, principally *Phoenix dactylifera*, which were chopped down following phytosanitary procedures for the reduction and prevention *of R. ferrugineus* with the assistance of the Department of Plant Protection, Palms and Dates Center [12]. Weevils were transported in plastic containers with palm tissues as a food supply in transit. Adults were fed on sugarcane plants (pieces, 4 gm each one) after emerging, and let out to oviposit in polycarbonate tissue culture bottles (350 mL, 75 mm, 109 mm height). In the laboratory, freshly hatched larvae were fed on sugarcane while being cultured in the plastic bottles that are mentioned above (10 larvae per each bottle). Insecticidal activity tests were conducted using larvae that were given untreated and treated sugarcane pieces. Adults were fed on sugarcane after emergence and then, they were moved to the polypropylene tissue culture bottles that are previously described. All of the insects were kept at 27 ± 2 °C at 74 ± 3% relative humidity.

### 2.2. Isolation of Rhizosphere Bacteria

Twenty soil samples were collected from date palm rhizosphere in Al Ahsa Oasis, Saudi Arabia. In order to grow bacterial isolates, a soil solution from each sample was well mixed by vortexing, and one loop was placed on nutrient agar (BD Difco™, BD Biosciences, Franklin Lakes, NJ, USA) plates (90 mm Ø) for further investigation. Incubation at 30 °C for 48 h was followed by storage at 4 °C until they were needed. For further isolation and to prevent the presence of background flora, individual colonies were picked up and resealed on new nutrient agar plates. The streaking process was repeated at least five times. Nutrient agar slants were used to cultivate 23 unidentified and purified bacterial strains. The 16S ribosomal DNA universal primers were used to identify the isolates [31]. PCR was performed as explained by Elsharkawy et al. [31]. Samples were properly prepared for dye terminator cycle sequencing using the BigDye Ready Reaction kit (Applied Biosystems, Foster City, CA, USA). The PCR conditions were 3 min at 94 °C, 24 cycles of 30 s at 94 °C for 30 s at 54 °C and 1 min at 72 °C, and then, 10 min at 72 and held at 4 °C. Applied Biosystems Genetic Analyzer which was installed with a 50 cm capillary array was used to conduct DNA sequencing. Sequence scanner software (Applied Biosystems) was utilized to read the sequencing data, and then nucleotide alignment blast was utilized to blast the unidentified sequences online on GenBank’s DNA sequences database. Neighbor-joining (NJ) algorithms were used to create phylogenetic trees from the alignment of the sequences utilizing MEGA 11.0.1 software [32]. Bootstrap analysis with 1000 repetitions of the same software was used to assess the dendrogram’s reliability. There were three separate replicates of this experiment.

### 2.3. Effect of the Bacterial Isolates on Larvae and Adults of RPW

After 12 h of incubation of the purified isolates at 30 °C, the inoculated Petri plates were counted for the number of colony forming units (CFU). Bacteria that were designated PDC-AHSAA 1, PDC-AHSAA 2, PDC-AHSA 3, and PDC-AHSA 4 demonstrated higher mortality in preliminary trials and were employed in subsequent trials. In diet integration bioassays, bacteria colony-forming units were counted after serial dilutions and adjusted using spectrophotometer. One centimeter of bacterial culture is used to quantify the optical density, which is the amount of light that is absorbed before it is detected by a photodiode. These isolates were tested on adult and younger larvae that were fed using a treated insect diet to determine its biological activity. The diet for the adults and larvae consisted of small, treated sugarcane pieces. The mortality was recorded after three weeks. The bioassay was applied using five different concentrations (1.56 × 10^7^, 3.39 × 10^8^, 8.59 × 10^9^, 4.27 × 10^10^, and 2.01 × 10^11^ CFU/mL). LC_50_ was determined using these rates after initial bracketing results. There was a bacteria-free control treatment. For each experiment, plastic boxes containing 40 RPW second-instar larvae or adults (females or males) for each treatment were starved for 3–4 h before their usage. Electronic balances were used to weigh the sugarcane pieces (before feeding) which were fed to each larva or adult every four days using the same concentration as for the tested bacteria. The sugarcane pieces were immersed for 20 min in either the bacterial suspensions or sterile distilled water before being provided to the larvae [17]. They were then left to air dry for 10 min at room temperature. There were four separate replicates of this experiment.

### 2.4. Effect of B. thuringiensis PDC-Ahsaa 1 on Larval Mortality 

For the experiment of mortality, different concentrations of Bt strain PDC-Ahsaa 1 (which showed good results in our preliminary experiments) were used against larvae and adults. Larvae and adults that did not respond to prodding with a needle were deemed to be dead. Following their exposure, the number of dead and living insects was reported every day for 21 days. Treatment of the feed at the same dose for larval bioassay was utilized for the adults. Four sets of ten duplicates for each concentration along with control were performed. For the purposes of this study, we investigated two types of mortality (mortality and corrected mortality) among larvae and adults. The mortality rate of larvae or adults was determined by dividing the total number of larvae that were tested by the number of dead larvae. The corrected mortalities of second-instar larvae were calculated using Abbott’s formula [33]. The effect of different concentrations of the strain PDC-AHSAA 1 on larval weight was measured. There were four separate replicates of this experiment.

### 2.5. Data Analysis 

Confidence intervals (95%), median lethal concentrations (LC_50_), and median lethal time (LT_50_) were calculated, as well as the means and standard errors (SE). Tukey’s studentized range test was used to detect the differences between treatment means. At a probability value of 5% (*p* ≤ 0.05), differences in means were considered significant. Analysis of variance (ANOVA) was utilized to statistically evaluate the data using the SPSS 17.0 for Windows (SPSS Inc., Chicago, IL, USA). 

## 3. Results

### 3.1. Isolation and Identification of Bacterial Isolates

**Figure 1:**
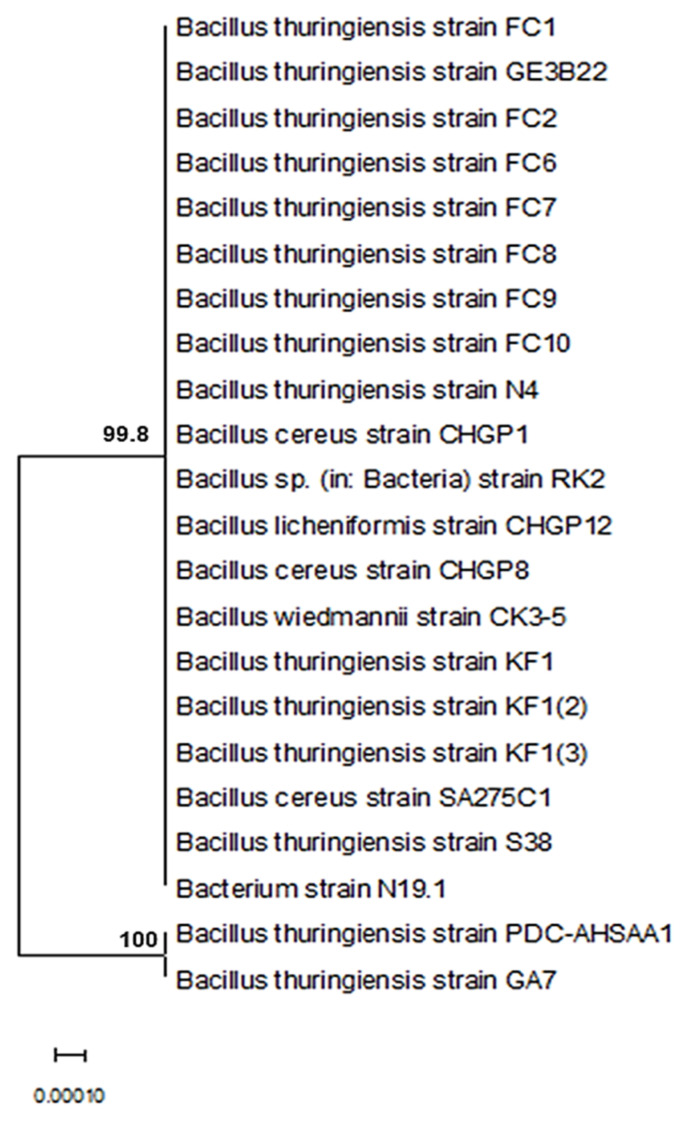
Phylogenetic dendrogram showing the position of *Bacillus thuringiensis* strain PDC-AHSAA1 among phylogenetic neighbors.

**Figure 2:**
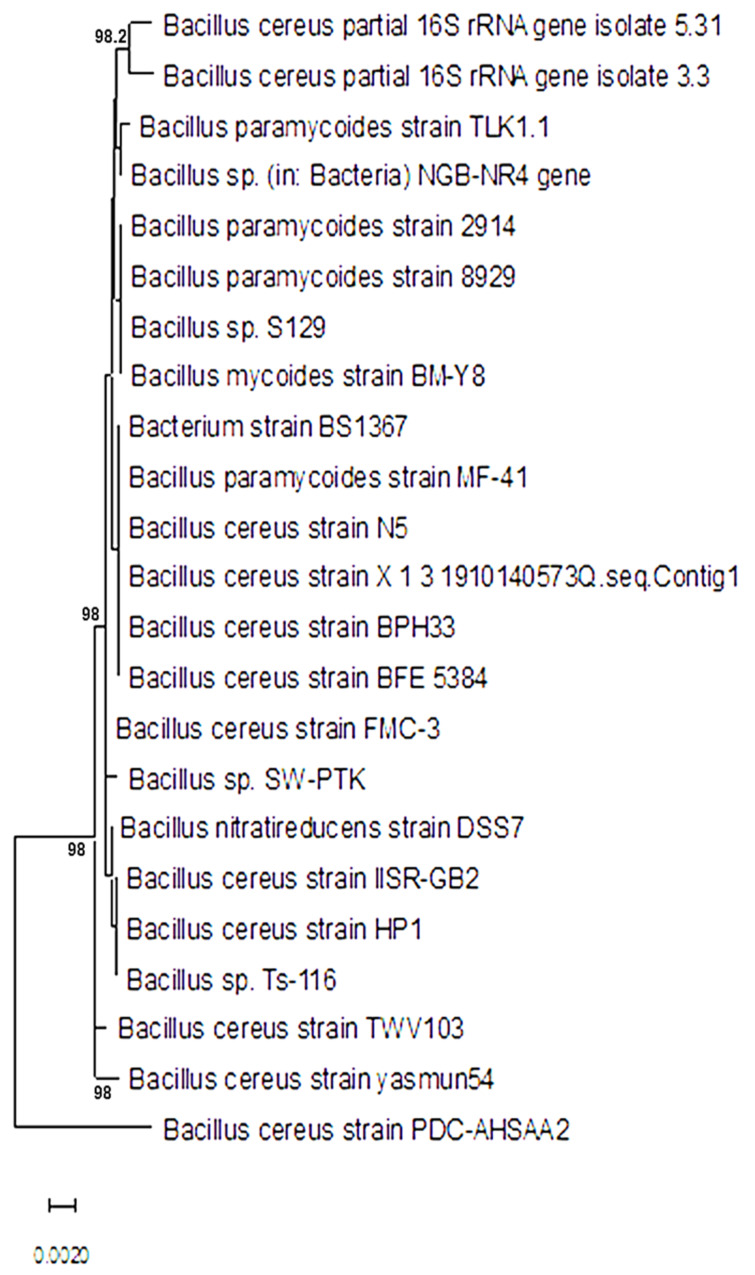
Phylogenetic dendrogram showing the position of *Bacillus cereus* strain PDC-AHSAA2 among phylogenetic neighbors.

**Figure 3:**
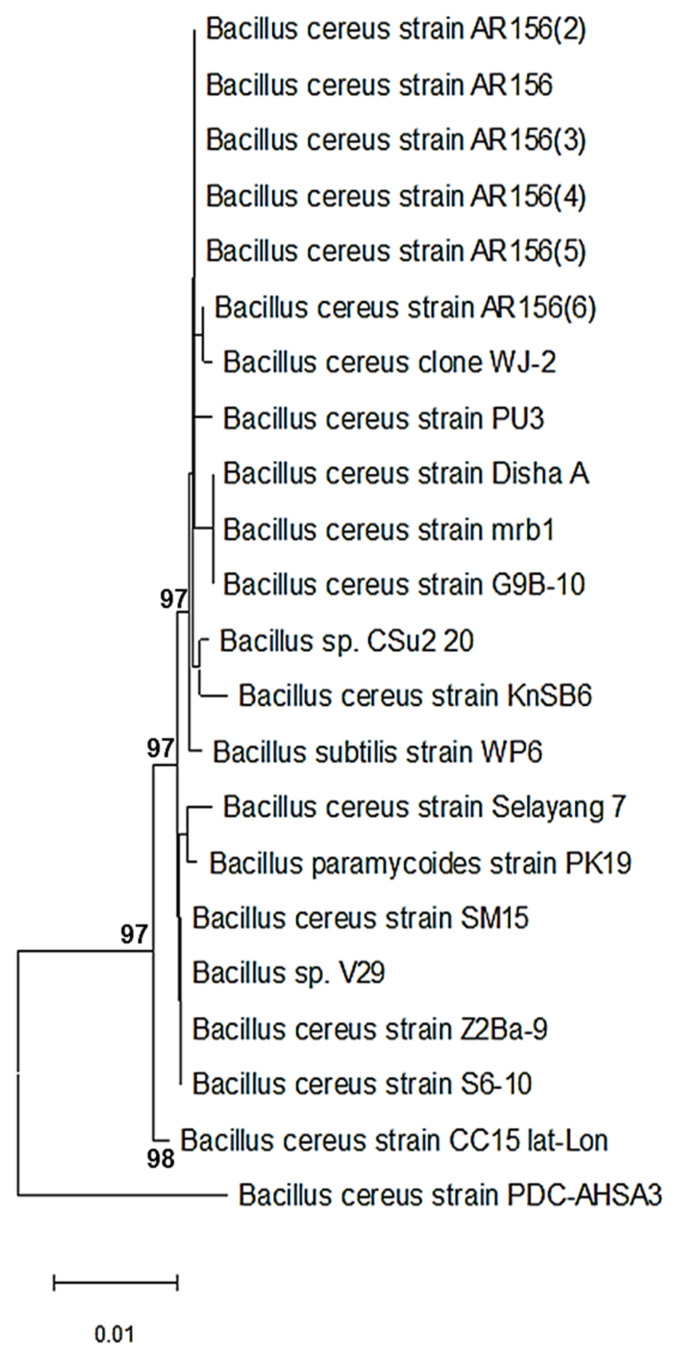
Phylogenetic dendrogram showing the position of *Bacillus cereus* strain PDC-AHSA3 among phylogenetic neighbors.

**Figure 4:**
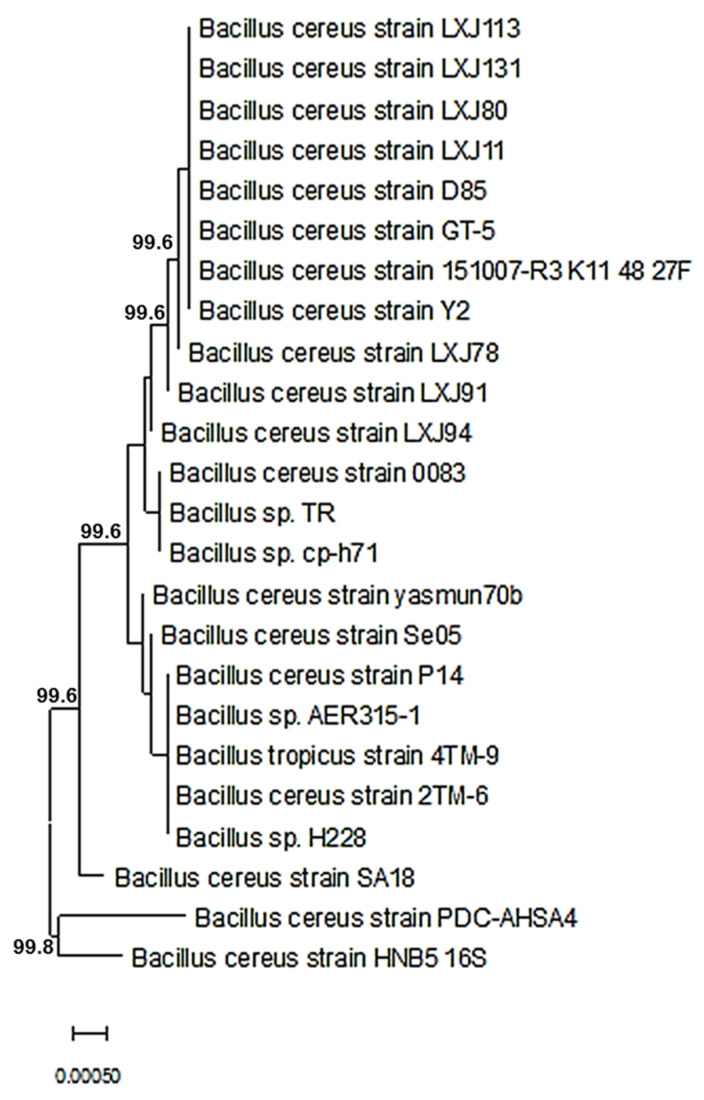
Phylogenetic dendrogram showing the position of *Bacillus cereus* strain PDC-AHSA4 among phylogenetic neighbors.

Twenty-three bacterial strains were purified and identified using the sequence of the 16S ribosomal RNA gene. The nucleotide database blasting using a nucleotide query technique demonstrated that the species *Bacillus* were the most prevalently discovered bacteria. The isolates were identified using amplicons that were amplified by the PCR method. The partial 16S rRNA gene sequences for PDC-AHSAA1, PDC-AHSAA2, PDC-AHSA3, and PDC-AHSA4 were submitted to NCBI with the respective accession numbers ON197897, ON197906, ON358125, and ON358129. The nucleotide alignment revealed that the PDC-AHSAA1 sequences were strongly connected with the *B. thuringiensis* strain GA7 from Turkey (100% similarity) when they were assessed according to the BLASTn (Figure 1), while the PDC-AHSAA2 sequences were connected with *B. cereus* from India (98.2% similarity) (Figure 2). The PDC-AHSA3 sequences were connected with the *B. cereus* strain CC15 lat-Lon from India (97.8% similarity) (Figure 3). Finally, the PDC-AHSA4 sequences were connected with the *B. cereus* strain HNB5 from China (99.8% similarity) (Figure 4). Among the isolated bacteria from the date palm rhizosphere, the *B. thuringiensis* strain PDC-AHSAA1, the *B. cereus* strain PDC-AHSAA2, the *B. cereus* strain PDC-AHSA3, and the *B. cereus* strain PDC-AHSA4 were selected for a further investigation. The preliminary bioassay results indicated that the second instar larvae and the adults of the RPW were extremely vulnerable to the isolates PDC-AHSAA1, PDC-AHSAA2, PDC-AHSA3, and PDC-AHSA4, more than any other isolates.

### 3.2. Effect of the Isolates on Larvae and Adults of RPW

The data showed that the *B. thuringiensis* strain PDC-AHSAA1 and the *B. cereus* strain PDC-AHSAA2 had LC_50_ values of 4.19 × 10^7^ and 4.96 × 10^7^ for the larvae, respectively. *B. thuringiensis* PDC-AHSAA1 was shown to be more effective than other isolates were (Table 1). Substantial differences in the 95 % fiducial limit between the LC_50_ values were regarded as evidence of there being significant differences. The adults were vulnerable to infection in the bioassays with sugarcane pieces that were contaminated by the bacteria. The potential of different bacterial isolates against the females was higher than it was for the males of the RPW. Both of the larvae and adults were affected by *B. thuringiensis* PDC-AHSAA1 more than they were by the *B. cereus* strains PDC-AHSAA2, PDC-AHSA3, and PDC-AHSA4 (Table 1). 

When it came to the larvae, the LT_50_ for the *B.* *thuringiensis* PDC-AHSAA1 and *B. cereus* PDC-AHSAA2 were 10 and 12 days, respectively. The LT_50_ of PDC-AHSAA1 were 13 and 11 days for males and females of the RPW, respectively (Table 2). The LT_50_ values of PDC-AHSA4 were 15, 17, and 16 days for the larvae, males, and females of the RPW, respectively (Table 2). The LT_50_ ranged from 10 to 17 days in the tested bacteria isolates. The results were maintained and extended to 21 days after treatment; however, they were not always statistically different (Table 2).

### 3.3. Effect of the Bacterial Isolates on Actual Larval Mortality

The mortality of the larvae, males, and females of the RPW that was caused by the treatment diet was high for the *B. thuringiensis* PDC-AHSAA1. The mortality rates were increased by increasing the concentration of the *B. thuringiensis* PDC-AHSAA1 in the treated diet. The mortality levels were higher in the treated females when they were compared to those that were reported in the males of the RPW (Table 3). Additionally, there were substantial differences in the overall corrected mortality data after feeding them with five different PDC-AHSAA1 concentrations. The concentration of 2.01 × 10^11^ CFU/mL had the most harmful impact in the RPW second-instar larvae, males, and females, resulting in mortality rates of 95.93, 90.46, and 93.98 %, respectively (Table 3). 

### 3.4. Effect of the Bacterial Isolates on Larval Weight

Over the course of the bioassays, the mortality of the RPW larvae that were fed a non-Bt control diet varied from 0% to 3%. Even at the lowest dose that was tested, the treated diet resulted in substantial death rates for the larvae, wherein the mortality rate rose to an extreme 95%. Table 4 shows that there were some impacts on the larval weight and behavior (a decrease in eating and mobility) which were detected. *B. thuringiensis* is predicted to affect the RPWs that are in older larval stages, and the dose-dependent mortality that we found for the larvae that were exposed was repeatable. It is possible that midgut damage or feeding inhibition occurred among the larvae who survived in the treatments, since the weight of the older instars was lower than that of the control ones.

## 4. Discussion

Twenty-three purified bacterial strains were isolated from the rhizosphere of date palm trees. The initial bioassays on the *R. ferrugineus* (RPW) larvae showed that some of the bacterial isolates had bioactivity that could be investigated. Four out of 23 bacterial isolates that were studied, PDC-AHSAA1, PDC-AHSAA2, PDC-AHSA3, and PDC-AHSA4, revealed that they had substantial harmful effects on the larvae and adults. The optical microscopy confirmed that the isolates’ morphological characteristics were rod-shaped, spore-forming, and Gram-positive bacteria. Both PDC-AHSAA1 and PDC-AHSAA2 were extremely effective biocontrol agents, causing death to the larvae and adults. Using a nucleotide query method, the sequences were blasted against a nucleotide database, and *Bacillus* was identified to be the most prevalent bacterial genera. Three isolates of *B. cereus* (PDC-AHSAA2, PDC-AHSA3, and PDC-AHSA4) and the *B. thuringiensis* strain PDC-AHSAA1 were successfully identified. *Bacillus thuringiensis* (Bt) is often referred to as a soil microorganism simply due to the fact that it has been isolated from this environment for several years [34,35]. Other researchers believe that the Bt spores may only be stored in the soil since they hardly germinate there and need certain nutrients and pH levels for that to occur [36,37]. The native microbiota and soil characteristics, including pH, humidity, mineral and organic matter concentrations, have an immediate impact on the Bt viability, thereby affecting its growth, sporulation, germination, and protein synthesis [37,38]. On the other hand, *B. cereus* (a taxon that is closely linked to Bt), which has shown the capacity to proliferate in non-sterilized soils, thereby expanding its population up to 20%, seems to be far less affected by these conditions [35]. However, under some circumstances, the Bt and *B. cereus* spores may germinate and develop successfully, such as in humid, nutrient-rich soils with pH levels that are close to neutral, even when other microbial communities are present [38]. The rhizosphere colonization by *B. cereus* and Bt has rarely been reported [39,40]. A Bt strain that was isolated from the rhizosphere of *Ficus doliaria* proved to be very toxic to the Blackfly larvae that were absent in the region [40]. *B. cereus* has also been reported to germinate and proliferate in the rhizosphere of plants [41]. The phenotypic identification of certain bacteria may be complicated by challenges and overlaps, however the 16S ribosomal DNA-based identification of unknown bacteria is an implemented method [42]. Universal primer mixtures may be used to amplify bacterial DNA [43]. The isolate sequences that were examined using BLASTn, a nucleotide alignment (bl2seq), had a high degree of alignment (100 %). An analysis of the 16S-rRNA sequences of each isolate was carried out using a neighbor-joining approach (NJ), which compared the sequences of the 16S rRNA with others in the database. The *Bacillus thuringiensis* strain GA7 (from Turkey), the *Bacillus cereus* strain (from India), the *Bacillus cereus* strain CC15 lat-Lon (from India), and the *Bacillus cereus* strain HNB5 (from China) were grouped with the isolates PDC-AHSAA1, PDC-AHSAA2, PDC-AHSA3, and PDC-AHSA4 with high-similarity levels (100, 98.2, 97.5, and 75%, respectively). High-similarity percentages indicated that the PCR products had been totally sequenced. This means that phenotypic characteristics at the species level were supported by the 16S rRNA sequencing-based genotyping. 

Several species of *Bacillus* have been reported to be entomopathogens and/or proteolytic bacteria that cause toxicity [40,41,44,45]. There are six closely related species in the *Bacillus cereus* group, including *B. thuringiensis*, *B. cereus*, *B. anthracis*, *B. mycoides*, *B. weihenstephanensis*, and *B. pseudomycoides*, which have been found to produce the paralytic toxins sphingomyelinase C and phospholipase C [46,47]. Among the isolated and identified bacteria, *B. thuringiensis* PDC-AHSAA1 has been demonstrated to have a more than a 95% larval mortality rate. Additionally, the potential of PDC-AHSAA1 on the RPW females was higher than it was for the RPW males. *B. thuringiensis* has long been regarded as the most important entomopathogenic bacterium in the control of insects. The oriental beetle, the European june beetle, the northern masked chafer, the shining leaf chafer beetle, the common European cockchafer, and the white grub (Coleoptera, Scarabaeidae) have all been effectively treated with the microbial control agents, *B. thuringiensis* or *B. cereus* [48,49,50,51]. The differences in the sensitivity across species are thought to be due to toxic protein receptors in the midgut and/or a complex of proteinases in the midgut. The protein is cleaved into a 60-kDa toxin by midgut enzymes which binds to the protein receptors on the brush-border membrane vesicles that are generated from the midgut epithelium. The toxin causes the swelling and the lysis of the gut epithelium by causing holes in the cell membrane [52]. Septicemia and starvation cause the insect’s death. The PDC-AHSAA1 application at the highest concentration that was tested (2.01 × 10^11^ CFU/mL) resulted in corrected mortality rates of 94.88, 93.14, and 89.71 % for the larvae, females, and males of the RPW, respectively. These results confirm that the larvae were more susceptible to PDC-AHSAA1 than the adults were, and also the males were more resistant than females were. Similarly, the larvae of the RPW were more sensitive to nanochitosan than the adults were, and the males were more resistant to it than the females were [53]. The longest survival period of the female and male that were treated with nanochitosan was 38.5 and 63 days, respectively, which was compared to that being 94.6 and 103 days in the control group, respectively. The larvae’s growth and development were affected by the PDC-AHSAA1-examined concentrations. Although the exact mechanism by which protease inhibitors increase Bt toxin activity is unknown, they speculate that they might inhibit specific gut proteases that would generally inactivate Bt toxins or limit proteolysis of membrane proteins, preventing the degradation of membrane-bound receptors, thus boosting their half-lives and ability to bind Cry toxins. The compound of the proteinases in the midgut of *Melolontha melolontha* (the European cockchafer) confers that they have a resistance to the Cry8C toxin [54]. The Cry8C toxin is degraded by the proteinases in vulnerable insects to a 67-kDa fragment that cannot be digested further or degraded into fragments of 10 kDa in resistant European cockchafers, thereby rendering it useless. Septicemia in *Lymantria dispar* (gypsy moth) and other lepidopteran larvae was caused by *Enterobacter* sp. in the majority of cases, but not in all of the species that were investigated [55,56]. The larvae and adults of the RPW appeared to be infected by the *B. cereus* and *B. thuringiensis* bacteria in this investigation. Our results indicate that the strain, *B. thuringiensis* PDC-AHSAA1, has a significant control impact on RPW larvae and adults.

## 5. Conclusions

Four isolated bacteria from the date palm rhizosphere were molecularly identified as *Bacillus thuringiensis* and *B. cereus*. They exhibited that they had the potential to be very effective against the RPW, and this study was conducted to reduce the consumption of synthetic chemical pesticides that are typically used to control the RPW populations. Due to their rapid death rate, these bacterial isolates may become potential entomopathogenic bacteria for controlling RPWs. Finally, additional research into the potential utility of Bt in managing RPWs under field conditions is needed. Additionally, the method of action and the molecular receptors that are implicated in causing insect death could be investigated in future studies. The study is one of the few to show the impact of entomopathogenic bacteria, *B. thuringiensis* and *B. cereus*, on RPW larvae and the first on female and male RPWs.

## Figures and Tables

**Table 1:** Insecticidal activity of *Bacillus thuringiensis* and *B. cereus* isolates against RPW larvae and adults after three weeks of bioassay.

RPW	Strain	LC_50_	95% FL	Slope ± SE
**Larvae**	**PDC-AHSAA1**	4.19 × 10^7^ b	4.02 × 10^6^–2.54 × 10^8^	0.91 ± 0.20
	**PDC-AHSAA2**	4.96 × 10^7^ b	4.91 × 10^6^–2.69 × 10^8^	1.37 ± 0.34
	**PDC-AHSA3**	2.83 × 10^8^ a	2.15 × 10^7^–2.79 × 10^9^	1.33 ± 0.31
	**PDC-AHSA4**	3.46 × 10^8^ a	3.13 × 10^7^–3.61 × 10^9^	1.12 ± 0.22
**Male**	**PDC-AHSAA1**	3.78 × 10^9^ b	3.66 × 10^8^–3.89 × 10^10^	1.27 ± 0.28
	**PDC-AHSAA2**	4.48 × 10^9^ b	4.15 × 10^8^–2.22 × 10^10^	1.19 ± 0.20
	**PDC-AHSA3**	4.99 × 10^10^ a	5.11 × 10^9^–2.58 × 10^11^	1.09 ± 0.19
	**PDC-AHSA4**	5.01 × 10^10^ a	5.05 × 10^9^–2.37 × 10^11^	1.67 ± 0.26
**Female**	**PDC-AHSAA1**	3.13 × 10^8^ b	3.12 × 10^7^–1.59 × 10^9^	1.57 ± 0.29
	**PDC-AHSAA2**	3.98 × 10^8^ b	3.65 × 10^7^–1.99 × 10^9^	1.29 ± 0.33
	**PDC-AHSA3**	4.80 × 10^9^ a	4.75 × 10^8^–2.65× 10^10^	1.12 ± 0.27
	**PDC-AHSA4**	4.82 × 10^9^ a	4.95 × 10^8^–2.74 × 10^10^	1.14 ± 0.31

**Table 2:** The impact of *Bacillus thuringiensis* and *B. cereus* isolates against RPW larvae and adults (males and females) after 21 days of the bioassay being performed.

RPW	Strain	LT_50_ (Days)	95% FL
**Larvae**	PDC-AHSAA1	10 × 10^8^ c *	8 × 10^4^–11 × 10^9^
	PDC-AHSAA2	12 × 10^8^ b	10 × 10^4^–13 × 10^9^
	PDC-AHSA3	15 × 10^8^ a	14 × 10^4^–16 × 10^10^
	PDC-AHSA4	15 × 10^8^ a	13 × 10^4^–16 × 10^10^
**Male**	PDC-AHSAA1	13 × 10^8^ c	11 × 10^4^–14 × 10^9^
	PDC-AHSAA2	15 × 10^8^ b	12 × 10^4^–15 × 10^9^
	PDC-AHSA3	17 × 10^8^ a	14 × 10^4^–18 × 10^9^
	PDC-AHSA4	17 × 10^8^ a	15 × 10^4^–19 × 10^8^
**Female**	PDC-AHSAA1	11 × 10^8^ c	9 × 10^4^–12 × 10^9^
	PDC-AHSAA2	13 × 10^8^ b	12 × 10^4^–15 × 10^9^
	PDC-AHSA3	16 × 10^8^ a	13 × 10^4^–17× 10^10^
	PDC-AHSA4	16 × 10^8^ a	14 × 10^4^–17 × 10^10^

* LT50′s reported in cfu’s/mL.

**Table 3:** The effects of *Bacillus thuringiensis* strain PDC-AHSAA1 concentrations on the mortality of RPW larvae and adults after 21 days of bioassay.

RPW	Strain Concentrations	Mortality (%)	Corrected Mortality (%)
**Larvae**	2.01 × 1011	95.93 ± 2.34 a	94.88 ± 2.24 a
	4.27 × 1010	91.85 ± 2.76 b	90.47 ± 2.36 b
	8.59 × 109	73.22 ± 1.89 c	72.20 ± 1.78 c
	3.39 × 108	71.98 ± 1.69 c	71.13 ± 1.34 c
	1.56×107	58.35± 1.42 d	57.97± 1.10 d
	Control	2.09 ± 1.13 e	na
**Male**	2.01 × 1011	90.46 ± 2.84 a	89.71 ± 2.31 a
	4.27 × 1010	81.59 ± 2.24 b	81.02 ± 2.11 b
	8.59 × 109	69.57 ± 1.59 c	68.92 ± 1.36 c
	3.39 × 108	63.51 ± 1.36 d	62.93 ± 1.29 d
	1.56×107	50.91 ± 1.09 e	50.03 ± 1.01 e
	Control	2.35 ± 1.10 f	na
**Female**	2.01 × 1011	93.98 ± 2.21 a	93.14 ± 2.13 a
	4.27 × 1010	88.46 ± 2.30 b	88.08 ± 1.86 b
	8.59 × 109	76.31 ± 1.47 c	75.92 ± 1.36 c
	3.39 × 108	70.29 ± 1.19 d	69.56 ± 1.15 d
	1.56×107	52.99 ± 2.01 e	51.71 ± 1.91 e
	Control	2.51 ± 1.19 f	na

na = not applicable.

**Table 4:** The effects of *Bacillus thuringiensis* strain PDC-AHSAA1 concentrations on the weight of RPW larvae after 21 days of bioassay.

Treatment	Weight (g) ± SE, (n = 30)
2.01 × 10^11^	2.53 ± 0.28 d
4.27 × 10^10^	2.68 ± 0.39 d
8.59 × 10^9^	3.35 ± 0.32 c
3.39 × 10^8^	3.49 ± 0.24 c
1.56 × 10^7^	3.96 ± 0.21 b
Control	4.43 ± 0.23 a

## Data Availability

Available upon request from the corresponding author.
